# Virtual Angle Boundary-Aware Particle Swarm Optimization to Maximize the Coverage of Directional Sensor Networks

**DOI:** 10.3390/s21082868

**Published:** 2021-04-19

**Authors:** Gong Cheng, Huangfu Wei

**Affiliations:** 1 School of Computer and Communication Engineering, University of Science and Technology Beijing, Beijing 100083, China; cgong1986@foxmail.com; 2 Shunde Graduate School, University of Science and Technology Beijing, Foshan 528300, China

**Keywords:** directional sensor networks, coverage optimization, boundary constraints, particle swarm optimization

## Abstract

With the transition of the mobile communication networks, the network goal of the Internet of everything further promotes the development of the Internet of Things (IoT) and Wireless Sensor Networks (WSNs). Since the directional sensor has the performance advantage of long-term regional monitoring, how to realize coverage optimization of Directional Sensor Networks (DSNs) becomes more important. The coverage optimization of DSNs is usually solved for one of the variables such as sensor azimuth, sensing radius, and time schedule. To reduce the computational complexity, we propose an optimization coverage scheme with a boundary constraint of eliminating redundancy for DSNs. Combined with Particle Swarm Optimization (PSO) algorithm, a Virtual Angle Boundary-aware Particle Swarm Optimization (VAB-PSO) is designed to reduce the computational burden of optimization problems effectively. The VAB-PSO algorithm generates the boundary constraint position between the sensors according to the relationship among the angles of different sensors, thus obtaining the boundary of particle search and restricting the search space of the algorithm. Meanwhile, different particles search in complementary space to improve the overall efficiency. Experimental results show that the proposed algorithm with a boundary constraint can effectively improve the coverage and convergence speed of the algorithm.

## 1. Introduction

Next-generation networks will support more devices, ushering in a new era of ubiquitous connectivity [1]. As an important part of wireless communication networks, Wireless Sensor Networks (WSNs) will be used more widely [2] and brings more convenient services for mobile Internet users. At present, WSNs are widely used in intrusion detection, environmental monitoring, intelligent transportation, industrial control and disaster management. In WSNs, the coverage reflects the ability of a sensor to monitor the target and obtain information, and it is an important index to judge the service quality of sensor networks. In recent years, with the combination of directional sensors [3] (image, video, infrared, ultrasonic sensor, etc.) and wireless communication technology, Directional Sensor Networks [4] (DSNs) have attracted a lot of attention. The sensing model of the directional sensor is a sector with the sensor position as the center of the circle, the furthest perceived distance as the radius, and the maximum perceived viewing angle as the flare angle. The sensing range of the directional sensor is limited and directional. These characteristics of the directional sensor make the coverage problem in DSNs more complex, so it is urgent to build a new problem model for it. Moreover, the coverage control algorithm based on the omnidirectional sensing model is difficult to be directly applied to the coverage problem of DSNs. To solve the coverage problem in DSNs, a new scheme that adapts to its characteristics needs to be designed.

Due to the limitation of cost, energy and integration requirements, the node is usually equipped with a single directional sensor to achieve network coverage. Combined with the 5G network scene, the application value of multi-functional directional sensor nodes is improved, making the coverage scheme with multiple directional sensors on a single node an option to improve network efficiency. The coverage optimization of DSNs can be achieved by adjusting the azimuth, flare angle, transmitting power, sensing radius, and other parameters of the directional sensor. In the actual environment, if the target area is larger, the number of coverage nodes increases correspondingly, which leads to a large number of adjustable parameters. The calculation of regional coverage consumes a lot of resources and time. Under the condition of minimal network resource consumption, the problem of providing coverage and capacity satisfying the demand of the target area has been proved to be NP-hard [5]. It is difficult to find the optimal solution in a short time. The existing network coverage optimization problems are mainly solved by machine learning training model [6,7] and random search [8,9,10]. However, these methods usually fail to take into account the structural redundancy among multiple sensors on the node, so such methods may lead to the reduction of the efficiency of the algorithm.

Since there is equivalent interchangeability between the directional sensors on the node, the problem of network coverage optimization can be solved by converting the original unconstrained sensor parameter optimization problem using Particle Swarm Optimization (PSO) algorithm. For multi-parameter joint debugging of fixed node multi-sensors, we proposed a new network coverage optimization scheme with a hybrid PSO algorithm based on Virtual Angle Boundary-aware (VAB-PSO) for DSNs. The VAB-PSO algorithm can use the existing search algorithm in the search space of eliminating redundancy to increase the efficiency of sensor multi-parameter joint coverage optimization in heterogeneous network scenarios. The main contributions can be summarized as follows.

1. The equivalent interchangeability of directional sensor coverage characteristics of the same node is proved from the perspective of mathematical theory.

2. A boundary constraint of eliminating redundancy scheme is constructed. The search space of the optimization algorithm is limited without reducing the coverage rate. Combined with the PSO algorithm, an efficient network coverage joint optimization algorithm for sensor azimuth and sensing range is proposed.

3. The proposed boundary constraint of eliminating redundancy scheme can not only combine the PSO algorithm but also be compatible with other optimization search algorithm to improve the efficiency of the algorithm.

The remainder of this paper is organized as follows—the related work of area coverage optimization scheme is presented in Section 2. The system model and problem formulation of the area coverage in DSNs are described in Section 3. In Section 4, the principle of sensor constraint transformation is proved and the VAB-PSO algorithm is designed. Section 5 deals with the simulation and comparison results, followed by the conclusion in Section 6.

## 2. Related Work

The coverage optimization problem of DSNs can be divided into the single-directional sensor and the multi-directional sensor according to the number of sensors on one node. According to the motion of the node, it can be divided into static and mobile sensors. According to different perspectives of algorithm optimization, it can be divided into distributed and centralized. Besides, there are also some DSNs that consider multiple coverage [11], probability coverage [12,13,14], graph theory [15,16,17], and other factors to achieve area coverage optimization under specific scenarios.

The single-directional sensor node realizes the area coverage of the whole network based on the coverage of the sensor in different directions at different times. With a combination of node mobility, the literature [18] proposed a two-phase coverage algorithm. In the first stage, the coverage direction of each sensor node is determined by the stepwise optimization method. In the second stage, the differential evolution algorithm is improved by using adaptive strategy and the virtual force algorithm is improved by using random control parameters to reduce the moving distance of the physical position of nodes. Similarly, literature [19] also maximizes the area coverage of the network through the rotation control of the sensor and the movement control of the node. However, the energy consumption of movable nodes is high, so most literature that adopts movable nodes need to study the coverage scheme and scheduling algorithm with low energy consumption. WSNs with stationary nodes have the advantages of low energy consumption and high network stability, but network flexibility is relatively insufficient. With the development of sensor node integration and energy-saving technology, the network value of a single node in video surveillance, industrial production, and other scenarios is constantly improving. Therefore, installing multiple directional sensors on a node can improve the efficiency of continuous network coverage under the condition of a limited cost increase. Combined with the advantages of stationary nodes, further optimization of network coverage can be realized by adjusting sensor sensing radius and other parameters.

The distributed characteristics of WSNs make it easy to deploy and organize themselves. To strengthen these advantages, network coverage optimization can adopt a distributed approach. The node determines the coverage scheme according to the neighbor node information within the sensing radius. The literature [20] proposed a learning algorithm based on distributed payment, which deployed and directed a specific number of directional sensor nodes. To maximize their coverage relative to their neighbors and achieve area coverage optimization. In the literature [21], an improved coverage method based on Delaunay triangulation to adjust sensor tilt was proposed to strengthen the coverage of weak spots. Each sensor independently makes a decision on the rotation direction of the sensor based on the local information of the neighboring sensor. However, the distributed coverage optimization method was prone to Sub-Optimization, which was not conducive to the overall optimization of the network. Centralized coverage optimization can obtain the global information of the network, and then carry out algorithm optimization on this basis to get the optimal coverage strategy. In reference [22], based on the characteristic of the periodic coverage of the area by the directional sensor, a time coverage model was established by minimizing the dark time of all targets, and a centralized Integer linear programming optimization algorithm and a distributed Initial orientation algorithm were proposed.

In reference [23], an Improved Adaptive Particle Swarm Optimization (IAPSO) algorithm was proposed to solve the problem of the blind area and overlapping coverage caused by the random deployment of directional sensor nodes. The area that cannot be covered by any sensors is called a blind area and if an area was covered by multiple sensors at the same time is called overlapping.The multi-objective optimization model was established to improve the coverage rate and reduce the redundancy rate. The design scheme improved the inertia weight of the traditional PSO algorithm. Compared with the traditional PSO algorithm, the IAPSO algorithm has a higher convergence ratio. This paper focuses on the coverage of the static multi-directional sensors network and adopts the centralized optimization algorithm to achieve efficient regional coverage. By analyzing the redundancy of the joint optimization search space of the sensor parameters of the node, the boundary constraint of eliminating the redundancy scheme is designed to limit the search space of the solution without reducing the optimization performance. The proposed network coverage optimization scheme takes into account the azimuth and sensing radius of the multi-directional sensors on the nodes. By using two kinds of optimal particle swarm and boundary particle swarm optimization, the convergence of the optimization algorithm is improved and the network coverage is improved in eliminating redundancy search space.

## 3. System Model and Problem Formulation

For the directional sensor nodes with relatively high deployment costs, installing multiple similar directional sensors on the same node can effectively reduce the total cost of the DSNs. According to the coverage requirements of the area, we adjust the angle and the sensing distance of each sensor so that the area covered by the DSNs can be as large as possible under the condition of a fixed number of sensors, which is the goal of our optimization. Meanwhile, considering the relationship between the energy consumption of the sensor and the sensing distance, reducing the energy consumption of the sensor as far as possible while ensuring the coverage area meets the requirements is another goal we need to optimize. In this section, we will model the system of coverage and energy consumption for DSNs, and translate these two optimization problems into a mathematical formula.

### 3.1. System Model

**Figure 1:**
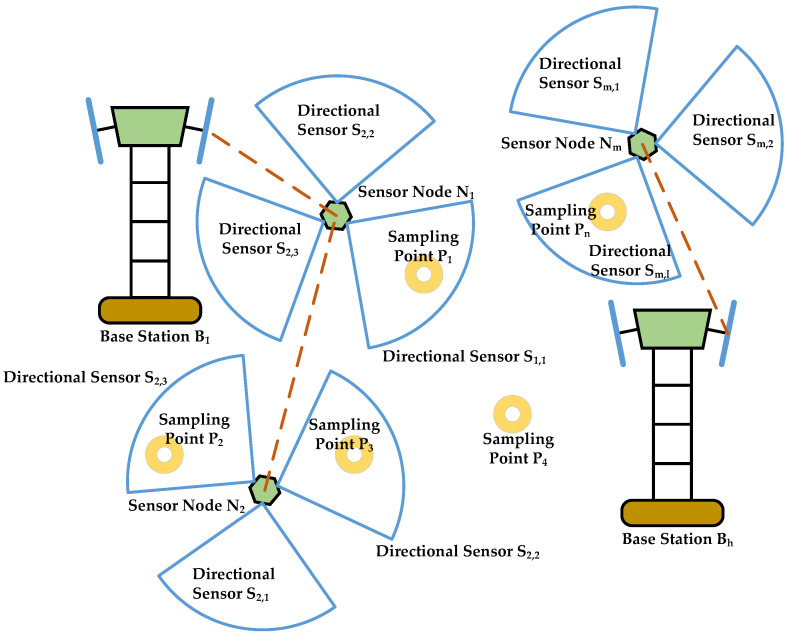
A coverage system model of Directional Sensor Networks (DSNs).

The areas covered by the DSNs are *R*. After the area is gridded, point set $P=\{P_{1},P_{2},.\ldots ,P_{n}\}$ is generated to determine the network coverage. There are *n* sampling points, where $P_{i}$ denotes the ith sampling point. The coverage rate of the entire area is evaluated based on the coverage of all sampling points. $N=\{N_{1},N_{2},\ldots ,N_{m}\}$ denotes the set of sensor nodes in the area and the number of nodes in the area is known to be *m*, where $N_{j}$ denotes the jth sensor node. and A certain number of directed sensors can be installed on the sensor node. Multiple directional sensors can be installed on sensor nodes. $S_{j,k}$ denotes the kth directional sensor on the jth sensor node. Then the set of directional sensors is denoted as $S=\{S_{1,1},S_{1,2},\ldots ,S_{m,l}\}$. $B=\{B_{1},B_{2},\ldots ,B_{m}\}$ represents the aggregation of base stations in the area, and *m* base stations are existing in the network. $B_{j}$ denotes the jth base station. The base station is responsible for transmitting the data acquired by the sensor node to the Internet. Here we assume that all sensor nodes can be directly connected to the base station or connected to the base station in a multi-hop manner through the sensor network. The diagram of the system is shown in Figure 1.

**Figure 2:**
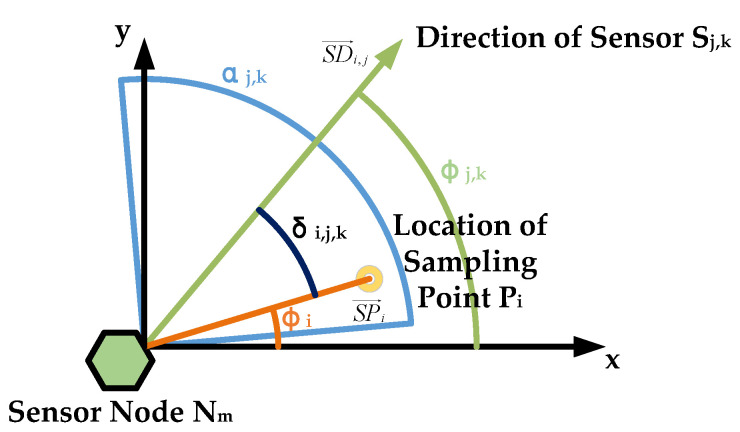
The coverage area of the directional sensor.

The nodes of the traditional omnidirectional sensor provide a circular coverage area, and the corresponding coverage area of the directional sensor presents a fan-shaped area, as shown in Figure 2. For the sensor $S_{j,k}$, $x_{j,k}$ and $y_{j,k}$ denote the *x* and *y* coordinates, respectively; $\phi_{j,k}$ denotes azimuth; $R_{j,k}$ denotes the sensing distance; $\alpha_{j,k}$ denotes the flare angle of the sensor. For sampling point $P_{i}$, $x_{i}$ and $y_{i}$ denote the *x* and *y* coordinates, respectively; $\phi_{i}$ denotes the angle of sampling point $P_{i}$ relative to the sensor $S_{j,k}$; $\delta_{i,j,k}$ denotes the included angle between the sampling point and the azimuth of the sensor. By solving the problem for optimizing the azimuth angle and sensing distance of the sensors on the node, the optimal coverage effect of the area is obtained and the energy consumption is minimized.

### 3.2. Problem Formulation

In this paper, the position of sensor nodes is assumed to be fixed, that is, the position optimization problem of sensor nodes is not discussed. Thus, the coordinates are regarded as known constants. This algorithm does not involve the location of nodes, and it is also not suitable for mobile nodes. Since the directional sensors installed on the sensor node adopt the same model, it means that the values of their induction angle α are the same, which are also regarded as constants. We need to jointly optimize two parameters of the sensor $S_{j,k}$, including azimuth $\phi_{j,k}$ and the sensing distance $R_{j,k}$. To better describe the problem, the following definitions are given.

**Definition** **1.** *The value of a function $f_{ps}\left(x_{i},y_{i},\phi_{j,k},R_{j,k}\right)$ is the coverage of the directional sensor $S_{j,k}$ to the sampling point $P_{i}$. The result is Boolean, with 1 indicating that it can be overridden and 0 indicating that it cannot be overridden. The meaning of the Formula (2) is to count the coverage of all directed sensors on the sampling point. The function $f_{ps}\left(x_{i},y_{i},\phi_{j,k},R_{j,k}\right)$ is given by:*

(1) $$f_{ps}\left(x_{i},y_{i},\phi_{j,k},R_{j,k}\right)=\left\{\begin{array}{c} 1,\;f_{dps}\left(x_{i},y_{i},\phi_{j,k},R_{j,k}\right)<D_{th}\;and\;f_{rps}\left(x_{i},y_{i},\phi_{j,k},R_{j,k}\right)<R_{th}\\ 0,\;others, \end{array}\right.$$

*where $D_{th}$ and $R_{th}$ respectively represent the threshold value of sensor coverage distance and angle. It can be seen from Figure 2 that $D_{th}=R_{j,k}$ and $R_{th}=\frac{\alpha }{2}$.*

**Definition** **2.** *The function $f_{p\mathrm{cov}}\left(x_{i},y_{i},\Phi ,R\right)$ represents the coverage at the sampling point $P_{i}$, which is described as:*

(2) $$f_{p\mathrm{cov}}\left(x_{i},y_{i},\Phi ,R\right)=\overset{m}{\underset{j=1}{⋃}}\overset{l}{\underset{k=1}{⋃}}f_{ps}\left(x_{i},y_{i},\phi_{j,k},R_{j,k}\right).$$

**Definition** **3.** *The function mix coverage rate $f_{\mathrm{cov}}\left(\Phi ,R\right)$ is given as:*

(3) $$f_{\mathrm{cov}}\left(\Phi ,R\right)=\frac{\sum_{i=1}^{n}f_{p\mathrm{cov}}\left(x_{i},y_{i},\Phi ,R\right)-0.05*\left(\sum_{i=1}^{n}\sum_{j=1}^{m}\sum_{k=1}^{l}f_{ps}\left(x_{i},y_{i},\phi_{j,k},R_{j,k}\right)-\sum_{i=1}^{n}f_{p\mathrm{cov}}\left(x_{i},y_{i},\Phi ,R\right)\right)}{n},$$

*where $\Phi =\{\phi_{1,1},\phi_{1,2},\ldots ,\phi_{m,l}\}$ denotes the set of all sensors’ azimuths and $R=\{R_{1,1},R_{1,2},\|,R_{m,l}\}$ denotes the set of all sensors’ sensing distance*.

**Definition** **4.** *$f_{dps}\left(x_{i},y_{i},\phi_{j,k},R_{j,k}\right)$ is the distance function from the sampling point $P_{i}$ to the directional sensor, which is given as:*

(4) $$f_{dps}\left(x_{i},y_{i},\phi_{j,k},R_{j,k}\right)=\sqrt{{\left(x_{i}-x_{j,k}\right)}^{2}+{\left(y_{i}-y_{j,k}\right)}^{2}}.$$

**Definition** **5.** *$f_{rps}\left(x_{i},y_{i},\phi_{j,k},R_{j,k}\right)$ is the included angle function between the sampling point $P_{i}$ and the azimuth of the directional sensor, which is given as:*

(5) $$f_{rps}\left(x_{i},y_{i},\phi_{j,k},R_{j,k}\right)=\arccos\frac{{\vec{SP}}_{i}\cdot {\vec{SD}}_{i,j}}{\left|{\vec{SP}}_{i}\right|\left|{\vec{SD}}_{i,j}\right|}$$

Ensure that the sampling point $P_{i}$ exists in the coverage area of the directional sensor $S_{j,k}$ given azimuth $\phi_{j,k}$ and sensing distance $R_{j,k}$.

Therefore, the constrained optimization problem can be formulated as:

**Problem:** The azimuth and sensing distance of the sensor are adjusted to maximize area coverage and minimize energy consumption.

(6) $$\begin{array}{cc} \; & \max\;f_{\mathrm{cov}}\left(\Phi ,R\right)\\ \; & s.t.\left\{\begin{array}{c} \phi_{j,k}\in \left[0,2\pi \right),j=1,2,...,m;k=1,2,\ldots ,l\\ R_{j,k}\in \left[R_{\min},R_{\max}\right),j=1,2,\ldots ,m;k=1,2,\ldots ,l, \end{array}\right. \end{array}$$

where the β is a constant defined by weighing the importance of the sub-objective. Through the optimization of constraint and the combination of the PSO algorithm, the proposed VAB-PSO scheme finally improves the overall efficiency of the algorithm.

## 4. An Area Coverage Optimization Scheme for DSNs

To solve the **Problem**, we design the VAB-PSO algorithm. The nodes use the VAB-PSO algorithm to jointly debug azimuth and sensing distance for full coverage of the target area.

### 4.1. Constraint Conversion

**Figure 3:**
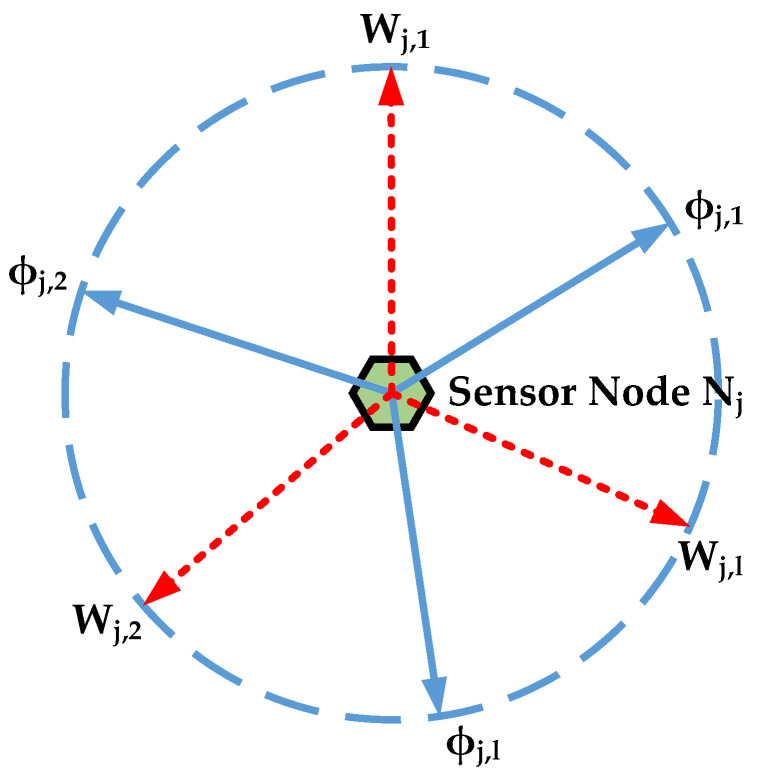
The location relationship between sensor azimuths and boundary constraint of the same node.

The location relationship between sensor azimuths and the boundary constraint of the same node is shown in Figure 3. There are *l* sensors on the node $N_{j}$, and their azimuths are $S_{j,1},S_{j,2},\ldots ,S_{j,l}$ respectively. Assume that the sensors have the same range. Since the coordinate position and the device type of the sensors are consistent, the radiation model of each sensor is the same. It can be considered that the sensors have isomorphism.

**Theorem** **1.** *In the same node, the new coverage rate obtained by exchanging the azimuths of any two sensors is the same as the original one.*

**Proof.**  Assume that the original azimuth of the sensors is $\Phi =\{\phi_{j,1},\phi_{j,2},\ldots ,\phi_{j,l}\}$ and the new azimuth is $\Phi^{\prime }=\left\{\phi {{}^{\prime }}_{j,1},\phi {{}^{\prime }}_{j,2},\ldots ,\phi {{}^{\prime }}_{j,l}\right\}$, where $\phi {{}^{\prime }}_{j,1}=\phi_{j,2}$,$\phi {{}^{\prime }}_{j,2}=\phi_{j,1}$, $\phi {{}^{\prime }}_{j,3}=\phi_{j,3}$, …, $\phi {{}^{\prime }}_{j,l}=\phi_{j,l}$.

Only the change of sensor azimuth is considered so that the range of all sensors are equal. Substituting the two sets of azimuths into the Formula (2),

(7) $$f_{p\mathrm{cov}}\left(x_{i},y_{i},\Phi^{\prime },R\right)=\overset{m}{\underset{j=1}{⋃}}\overset{l}{\underset{k=1}{⋃}}f_{ps}\left(x_{i},y_{i},\phi {{}^{\prime }}_{j,k},R_{j,k}\right).$$

Since $\phi {{}^{\prime }}_{j,1}=\phi_{j,2}$ and $\phi {{}^{\prime }}_{j,2}=\phi_{j,1}$, substituting them in the Formula (8),

(8) $$f_{ps}\left(x_{i},y_{i},\phi {{}^{\prime }}_{j,1},R_{j,k}\right)=f_{ps}\left(x_{i},y_{i},\phi_{j,2},R_{j,k}\right)$$

(9) $$f_{ps}\left(x_{i},y_{i},\phi {{}^{\prime }}_{j,2},R_{j,k}\right)=f_{ps}\left(x_{i},y_{i},\phi_{j,1},R_{j,k}\right).$$

Substituting Formula (9) and (10) in (8),

(10) $$\overset{m}{\underset{j=1}{⋃}}\overset{l}{\underset{k=1}{⋃}}f_{ps}\left(x_{i},y_{i},\phi {{}^{\prime }}_{j,k},R_{j,k}\right)=\overset{m}{\underset{j=1}{⋃}}\overset{l}{\underset{k=1}{⋃}}f_{ps}\left(x_{i},y_{i},\phi_{j,k},R_{j,k}\right).$$

Then,

(11) $$f_{p\mathrm{cov}}\left(x_{i},y_{i},\Phi^{\prime },R\right)=f_{p\mathrm{cov}}\left(x_{i},y_{i},\Phi ,R\right)$$

Finally obtaining,

(12) $$f_{\mathrm{cov}}\left(\Phi ,R\right)=f_{\mathrm{cov}}\left(\Phi^{\prime },R\right).$$

□

**Theorem** **2.** *In the same node, the new coverage rate obtained by sorting the sensors is the same as the original one.*

**Proof.**  Assume that the original azimuth of the sensors is $\Phi =\{\phi_{j,1},\phi_{j,2},\ldots ,\phi_{j,l}\}$, where $\phi_{j,1}<\phi_{j,3}<\phi_{j,2}<\ldots <\phi_{j,l}$. The new azimuth is $\Phi^{\prime }=\left\{\phi {{}^{\prime }}_{j,1},\phi {{}^{\prime }}_{j,2},\ldots ,\phi {{}^{\prime }}_{j,l}\right\}$, where $\phi {{}^{\prime }}_{j,1}=\phi_{j,1}$, $\phi {{}^{\prime }}_{j,2}=\phi_{j,3}$, $\phi {{}^{\prime }}_{j,3}=\phi_{j,2}$, ..., $\phi {{}^{\prime }}_{j,l}=\phi_{j,l}$. The parameters of the new sensor are obtained by interchanging $\phi_{j,2}$ and $\phi_{j,3}$ of the original sensor parameter. Therefore, $\phi {{}^{\prime }}_{j,1}<\phi {{}^{\prime }}_{j,2}<\phi {{}^{\prime }}_{j,3}<\ldots <\phi {{}^{\prime }}_{j,l}$.

According to Theorem 1, it can be concluded that the coverage of the node is invariant after exchanging two azimuths arbitrarily. One solution to the sorting problem is to exchange elements based on size. Therefore, sorting by the angle size can be completed by exchanging azimuths reasonably. The coverage rate corresponding to the new azimuths after sorting is the same as the coverage before sorting. □

**Theorem** **3.** *Boundary constraints are added between adjacent azimuths of the same node. After limiting the value range of the azimuth angle, the solution space obtained is equivalent to the original problem.*

**Proof.**  Assume that the original azimuth of the sensors is $\Phi =\{\phi_{j,1},\phi_{j,2},\ldots ,\phi_{j,l}\}$. The azimuths of the new sensors are the results after sorting the azimuths of the original sensors. Boundary constraints are $W_{j,1},W_{j,2},\ldots ,W_{j,l}$ and satisfy $\phi {{}^{\prime }}_{j,l}-2\pi <W_{j,1}<\phi {{}^{\prime }}_{j,1}<W_{j,2}<\phi {{}^{\prime }}_{j,2}<...<W_{j,l}<\phi {{}^{\prime }}_{j,l}<W_{j,1}+2\pi$.

According to Theorem 2, the coverage rates of $\phi^{\prime }$ and ϕ are the same. In other words, after sorting the original parameters and adding boundary constraints between adjacent sensors, the initial solution remains unchanged.

For $\phi {{}^{\prime }}_{j,2}$, after adding the boundary constraint, $W_{j,2}<\phi {{}^{\prime }}_{j,2}<W_{j,3}$. In the original problem, $\phi {{}^{\prime }}_{j,2}$ satisfies $0<\phi {{}^{\prime }}_{j,2}<2\pi$, that is, there is no constraint on azimuth. Since the position $W_{j,3}$ of the boundary constraint is between $\phi_{j,2}$ and $\phi {{}^{\prime }}_{j,3}$ can be moved, the value space of $\phi {{}^{\prime }}_{j,2}$ in the counterclockwise direction theoretically is $W_{j,2}<\phi {{}^{\prime }}_{j,2}<\phi {{}^{\prime }}_{j,3}$. When $\phi {{}^{\prime }}_{j,2}$ need to satisfy $\phi {{}^{\prime }}_{j,2}>\phi {{}^{\prime }}_{j,3}$, we can exchange $\phi {{}^{\prime }}_{j,2}$ and $\phi {{}^{\prime }}_{j,3}$ based on **Theorem 1**. The exchanged problem is equivalent to the original problem, that is, the actual value space $\phi {{}^{\prime }}_{j,2}$ can increase the value space $\phi {{}^{\prime }}_{j,3}$. The solution space $\phi {{}^{\prime }}_{j,2}$ becomes $W_{j,2}<\phi {{}^{\prime }}_{j,2}<W_{j,1}$. Similarly, it can be deduced that the equivalent value space $\phi {{}^{\prime }}_{j,2}$ in the clockwise direction is $W_{j,1}<\phi {{}^{\prime }}_{j,2}<W_{j,3}$. In this case, the solution spaces of the two directions are coincident and $0<\phi {{}^{\prime }}_{j,2}<2\pi$ can be realized. After adding the boundary constraint, the solution space is the same as the original problem. The same goes for $\phi {{}^{\prime }}_{j,1}$ to $\phi {{}^{\prime }}_{j,l}$. □

Therefore, the original optimization problem is transformed into

(13) $$\begin{array}{cc} \; & \max\;f_{\mathrm{cov}}\left(\Phi ,R\right)\\ \; & s.t.\left\{\begin{array}{c} \phi_{j,k}-2\pi <W_{j,1}<\phi_{j,1}<W_{j,2}<\phi_{j,2}<\ldots <W_{j,k}<\phi_{j,k}<W_{j,1}+2\pi ,\\ j=1,2,\ldots ,m;k=1,2,\ldots ,l\\ R_{j,k}\in \left[R_{\min},R_{\max}\right),j=1,2,\ldots ,m;k=1,2,\ldots ,l. \end{array}\right. \end{array}$$

Based on the isomorphism of the sensor, the boundary constraint to limit the sensor range can be added between adjacent sensors on the same node, and the solution space remains unchanged. The range of search is effectively reduced, so the redundant parts in solution space are removed and the search speed is accelerated.

### 4.2. VAB-PSO Algorithm

For network coverage optimization, the PSO algorithm can be optimized by applying the boundary constraint of eliminating redundancy techniques to limit the searching range. The boundary condition is used to deal with the particle movement beyond the limit of boundary constraint [24,25]. At the same time, when the algorithm is updated iteratively, the boundary constraint is updated to assist the next round of search. To increase the flexibility of boundary constraint, a new VAB-PSO algorithm is proposed to add a class of particles with optimal boundary constraint (BCP) to the class of particles used for optimal coverage search (CSP). Standard PSO algorithm searches are performed by each particle in the population. To find the local optimal solution, the search direction of each particle is determined jointly by the historical best position found by the particle (pbest) and the historical best position of the population (gbest), which is described as:

(14) $$\mathrm{pbest}\left(o,t\right)=\arg\max\left[f\left(L_{o}\left(t\right)\right)\right],\;o\in \left\{1,2,\ldots ,N_{o}\right\},$$

(15) $$\mathrm{gbest}\left(t\right)=\arg\max[f\left(L_{o}\left(t\right)\right)],$$

where *o* denotes the index of particles, $N_{o}$ denotes the total number of particles, *t* denotes the current number of iterations, *f* denotes the fitness function and *L* denotes the position of the particle. Update relationship between velocity *V* and position *L* is given as:

(16) $$V_{o}\left(t+1\right)=\omega V_{o}\left(t\right)+c_{1}r_{1}\left(\mathrm{pbest}\left(o,t\right)-L_{o}\left(t\right)\right)+c_{2}r_{2}\left(\mathrm{gbest}\left(t\right)-L_{o}\left(t\right)\right)$$

(17) $$L_{o}\left(t+1\right)=L_{o}\left(t\right)+V_{o}\left(t+1\right)$$

where $r_{1}$ and $r_{2}$ are inertial weights, used to balance global and local search, and are random variables uniformly distributed in the range of [0, 1]. $c_{1}$ and $c_{2}$ are constant parameters with positive values called acceleration coefficients.

Some classical parameters are ω=0.7, $c_{1}=c_{2}=1.5$; ω=0.8, $c_{1}=2.1$, $c_{2}=1.5$; ω=0.6, $c_{1}=c_{2}=3.0$.

According to the Theorems in the previous section, the coverage function is adjusted appropriately and a subparameter *W* is added to represent the boundary constraint. Optimization problems applicable to PSO are translated into:

(18) $$\begin{array}{cc} \; & \max\;f_{\mathrm{cov}}\left(\Phi ,R,W\right)\\ \; & s.t.\left\{\begin{array}{c} \phi_{j,k}-2\pi <W_{j,1}<\phi_{j,1}<W_{j,2}<\phi_{j,2}<...<W_{j,k}<\phi_{j,k}<W_{j,1}+2\pi ,\\ j=1,2,\ldots ,m;k=1,2,\ldots ,l\\ R_{j,k}\in \left[R_{\min},R_{\max}\right),j=1,2,\ldots ,m;k=1,2,\ldots ,l. \end{array}\right. \end{array}$$

$f_{\mathrm{cov}}\left(\Phi ,R,W\right)$ is a function to evaluate the coverage rate with the PSO algorithm, where *W* does not participate in the calculation of coverage and is only used to limit the moving range of particles.

Suppose $N_{u}$ particles are searching for angles, and the velocity update formula of the uth particle is

(19) $$V_{u}\left(t+1\right)=\omega_{u}V_{u}\left(t\right)+c_{u1}r_{u1}\left(\mathrm{pbest}\left(u,t\right)-L_{u}\left(t\right)\right)+c_{u2}r_{u2}\left({\mathrm{gbest}}_{u}\left(t\right)-L_{u}\left(t\right)\right).$$

Suppose $N_{w}$ particles are searching for boundary constraints, and the velocity update formula of the wth particle is

(20) $$V_{w}\left(t+1\right)=\omega_{w}V_{w}\left(t\right)+c_{w1}r_{w1}\left(\mathrm{pbest}\left(w,t\right)-L_{w}\left(t\right)\right)+c_{w2}r_{w2}\left({\mathrm{gbest}}_{w}\left(t\right)-L_{w}\left(t\right)\right).$$

The treatment methods for errant particles by boundary conditions are selected as [25]. The search for the maximum coverage rate is completed through the iterative update of the two kinds of particle swarms. Finally, the optimal solution of azimuth and title is obtained, and the VAB-PSO algorithm is designed.

### 4.3. Realization of the Area Coverage Optimization Scheme

First, the sensor azimuths on the same node are sorted in the initialization of the VAB-PSO algorithm. The initial values of boundary constraints are generated between adjacent sensors. The initial particle swarm is then generated within the range of each parameter. After that, the fitness of each particle swarm is calculated and iterated. Then we determine the relationship between the position and the boundary constraint. When boundary crossing occurs, the treatment methods for errant particles by boundary conditions are selected as [25]. The boundary constraint position corresponding to the angles found by CSP is taken as the optimal position for searching BCP, and around the update of BCP is carried out. The search for the maximum coverage is completed by the cross iterative update of two-particle swarms. Finally, the optimal solution of azimuth and sensing distance is obtained to achieve the target coverage of the area. The pseudocode of the VAB-PSO algorithm is described in Algorithm 1.

**Algorithm 1:** VAB-PSO algorithm

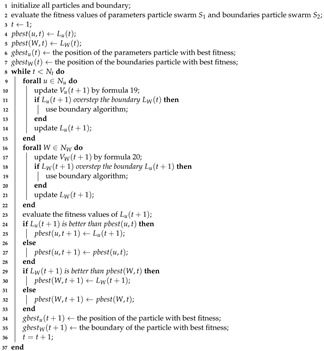



## 5. Simulations and Comparisons

In this section, the performance of the proposed algorithm will be evaluated in the mathematical model through experimental simulation. According to the proposed hybrid optimization strategy of two kinds of particle swarms, the coverage azimuth and the sensing distance of multi-directional sensors on nodes are optimized and solved. Firstly, the solution space of sensor parameters is limited by the optimization results of boundary particle swarm optimization, and then the optimal solution of network coverage is obtained in the solution space of each sensor parameter through optimizing particle swarm. Achieve the maximum area covered under the condition of low energy consumption. In this paper, the IAPSO algorithm is selected as a comparison scheme. Experimental scenes are divided into algorithm simulations under ideal terrain and actual environment data.

### 5.1. Parameters Setting

The simulation experiment is implemented in Python and running on the platform Microsoft Windows 10. In both experimental scenarios, the number and location of sensor nodes are fixed and known. The number of directional sensors installed on each node is adjustable, and the influence of 3 or 4 sensors installed on network coverage is considered in the experiment. The parameters are described below in Table 1. When the optimization variables exceed their value range, the absorbing method and damping methods are respectively used for boundary treatment. All experimental results of the algorithms are obtained by using the same random number seed for multiple simulations.

### 5.2. The Ideal Scene

**Figure 4:**
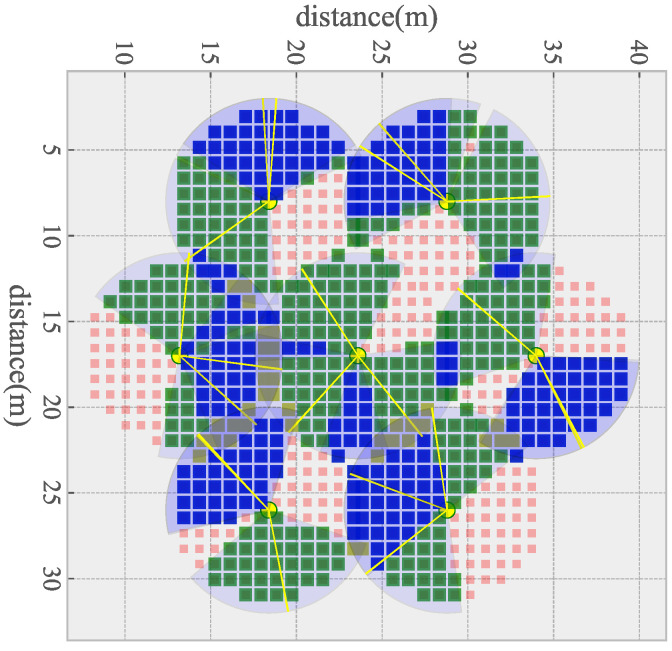
The initial coverage of an idea map.

The initial coverage of an ideal DSN deployment scene is shown in Figure 4. The square represents the sampling point, the red one represents that the point is not covered, the green one represents that the point is covered by one sensor, the blue one represents that the point is covered by two sensors and the gray one represents that the sampling point is covered by three or more sensors. The yellow dot represents the position of the sensor, and the yellow line segment represents the covering direction of the sensor. It can be seen from Figure 4 that, under initial conditions, a relatively close azimuth of the sensor on the same node will lead to a low coverage rate.

As the optimization effect of the algorithm will change with the parameters set during simulation, the performance of the algorithm should be close to optimal by selecting appropriate parameters. Among all kinds of parameters, the swarm size is directly related to the computation burden of the algorithm. After determining the optimal swarm size of different algorithms, the speed of other parameter optimization can be accelerated.

**Figure 5:**
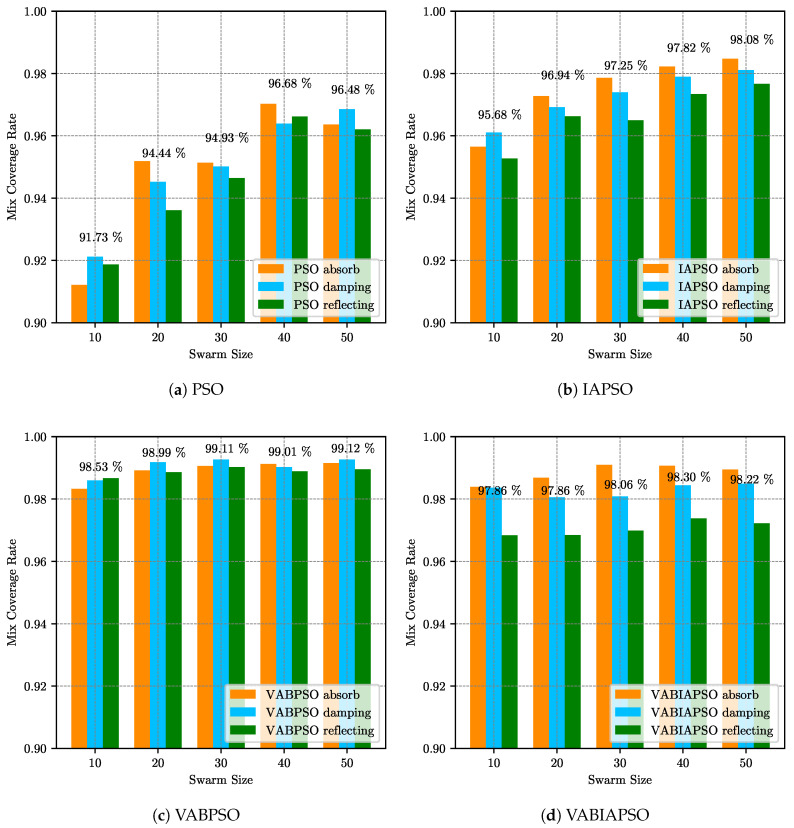
The mix coverage of the four algorithms under different swarm sizes in the ideal scene.

Figure 5 describes the mix coverage rates of the PSO, IAPSO, VABPSO and VABIAPSO algorithms when the swarm size is (10, 20, 30, 40, 50) respectively. The final coverage effects of three different boundary processing methods which are absorbing, damping and reflecting for each algorithm are tested under the same swarm size, absorb, and reflect realization. The results are differentiated in different colors. At the top of the histogram, the mean values of the mix coverage rates for swarm sizes are numerically plotted.

As can be seen from Figure 5a, the average mix coverage rate of the standard PSO algorithm increases gradually with the increase of swarm size. When the swarm size is 40, the average composite coverage rate reaches the maximum. With the further increase in the population, the value decreases slightly. Therefore, the optimal effect of the algorithm can be achieved by selecting the swarm size of 40 and absorbing the boundary treatment method as the parameter of the standard PSO algorithm in the ideal map. As shown in Figure 5b, when the swarm size of the IAPSO algorithm increases from 40 to 50, the average mix coverage rate improves by less than 0.3%, and the effect is basically the same. In order to ensure the operational efficiency of the algorithm, the swarm size of 40 should be selected as the best parameter of the algorithm, and the boundary treatment method is chosen as the absorb. According to Figure 5c, the optimal swarm size in the VABPSO algorithm could be set as 20, and the boundary processing method is chosen as damping. For the VABIAPSO algorithm in Figure 5d, although the average mix coverage rate is not the maximum when the swarm size is 30, the boundary treatment method has a great influence on this algorithm. Finally, the optimal parameters are selected as the swarm size of 30 and the absorbing boundary treatment method.

**Figure 6:**
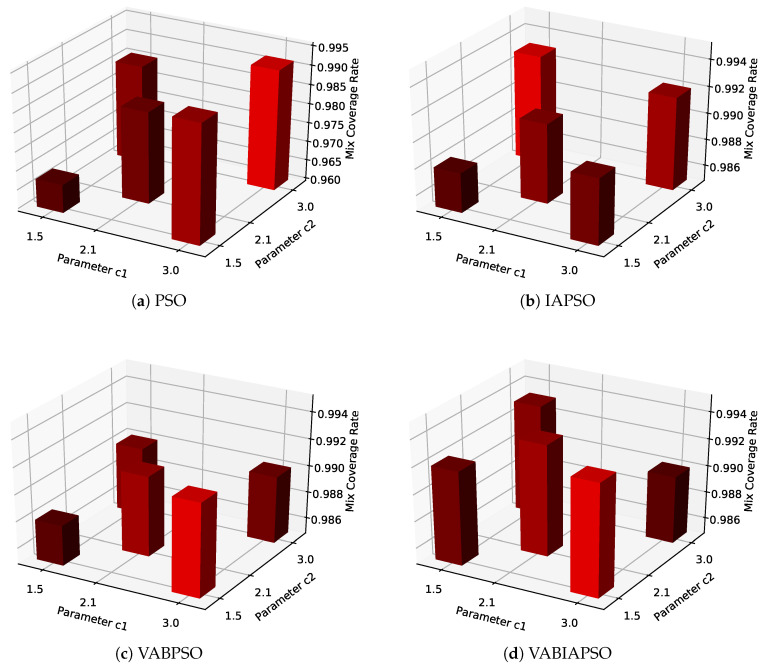
The mix coverage of the four algorithms under different values of $C_{1}$ and $C_{2}$ in the ideal scene.

After determining the optimal swarm size and boundary treatment method of each algorithm, $C_{1}$ and $C_{2}$ parameters of the algorithms are further determined to optimize the algorithm performance. The mix coverage obtained by the four algorithms under the combination of different parameters is shown in Figure 6.

The value combinations of $C_{1}$ and $C_{2}$ are shown in the Figure 6 as values on the *X*-axis and *Y*-axis (1.5, 2.1, 3.0) respectively. The *Z*-axis histogram shows the maximum mix coverage rate that the algorithm can achieve under this combination of values. According to the mix coverage, $C_{1}$ = 3.0 and $C_{2}$ = 3.0 in Figure 6a are selected as the best parameters of the standard PSO algorithm, $C_{1}$ = 1.5 and $C_{2}$ = 3.0 in Figure 6b are selected as the best parameters of IASPO algorithm, $C_{1}$ = 3.0 and $C_{2}$ = 1.5 in Figure 6c,d are selected as the best parameters of VABPSO and VABIAPSO algorithms. Each diagram is highlighted in bright red. It is worth noting that in the VABIAPSO algorithm, the effect of multiple parameters combination is very close and all are relatively excellent, indicating that the algorithm has strong adaptability.

**Figure 7:**
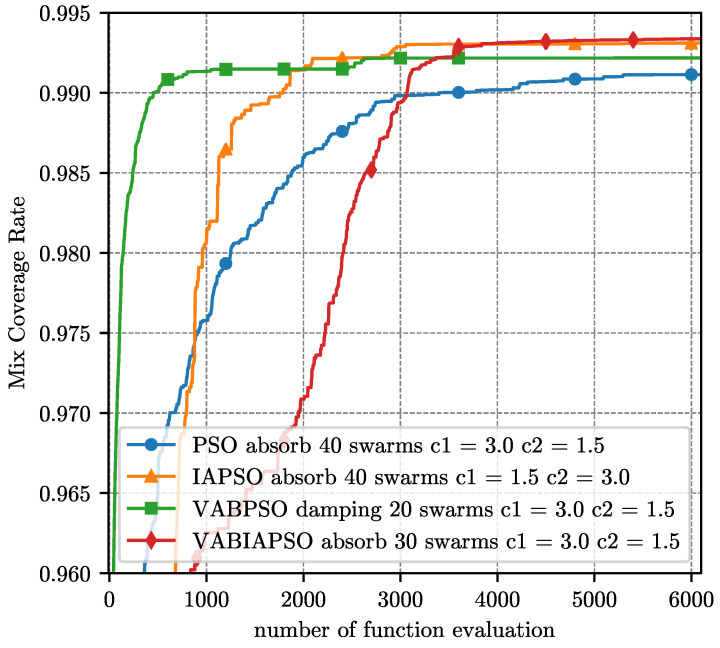
Performance comparison of four algorithms under the optimal parameters of the ideal scene.

The optimal parameters determined by the above experiment are substituted into the four algorithms respectively, and the final coverage performance results under the ideal scene are compared as shown in Figure 7. Considering that the swarm sizes in the optimal parameters of different algorithms are not consistent, here we evaluate the performance of the algorithm according to the number of function evaluation (FE) method.

The final mix coverage of the four algorithms is close to 100%, which shows the effectiveness of the algorithms. In the initial stage of the IAPSO algorithm, the convergence speed is relatively slow compared with the standard PSO algorithm. When the IAPSO algorithm runs nearly 900 operations, the convergence speed exceeds the standard PSO. Finally, it converges around 3000 operations to achieve the second-highest mix coverage rate. The VABPSO algorithm has the fastest convergence speed, making the mix coverage rate over 99% in 500 operations. The convergence is completed in 1000 operations. The convergence speed of the VABIAPSO algorithm is not fast, and it does not exceed the standard PSO algorithm until about 3000 operations. However, the maximum mix coverage rate is obtained, which indicates that the algorithm has a strong searching ability despite its slow convergence speed.

**Figure 8:**
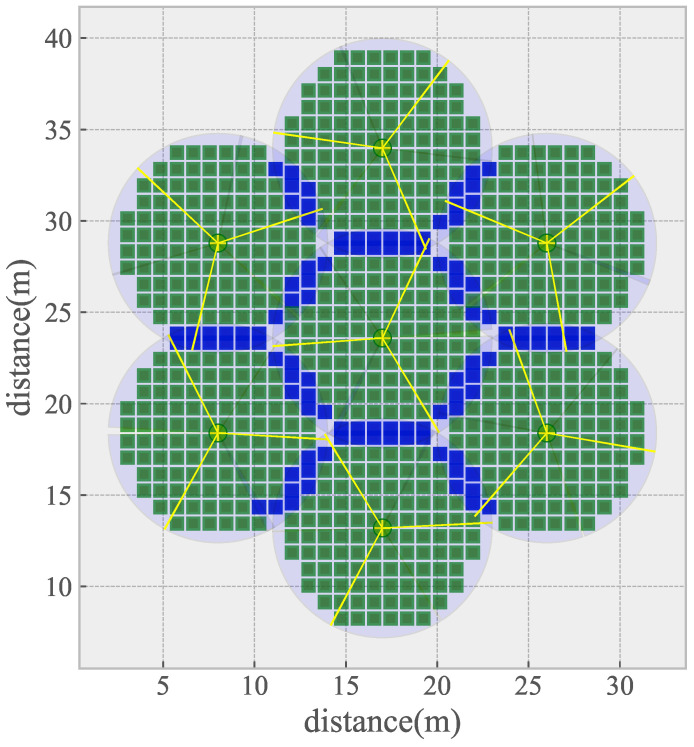
The coverage of the ideal scene after algorithm optimization.

After optimization, the coverage effect of sampling points in the ideal scene is shown in Figure 8. The final effect of the four algorithms is consistent. It can be seen that these algorithms can solve the coverage optimization problem in the ideal scene. The difference between algorithms is reflected in the convergence speed, that is, the length of time to realize the optimal coverage is different.

### 5.3. The Real Scene

**Figure 9:**
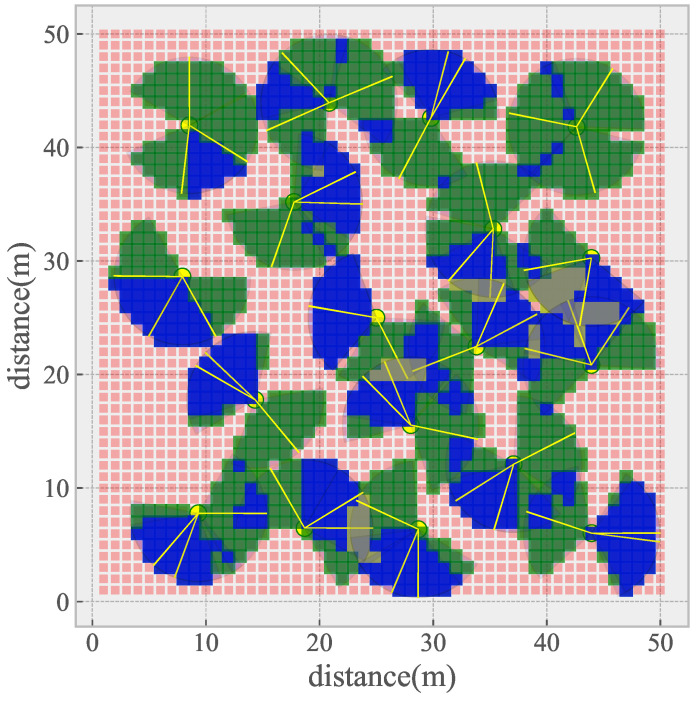
Initial coverage of a real DSN deployment scene.

In the real scene, the initial coverage of sampling points in the region is shown in Figure 9. Different from the ideal scene, in the real scene, the overlapping area between sensor nodes close to each other is more random, and the coverage relationship between multiple nodes is more complex, so it is difficult to simply get the best coverage scheme.

**Figure 10:**
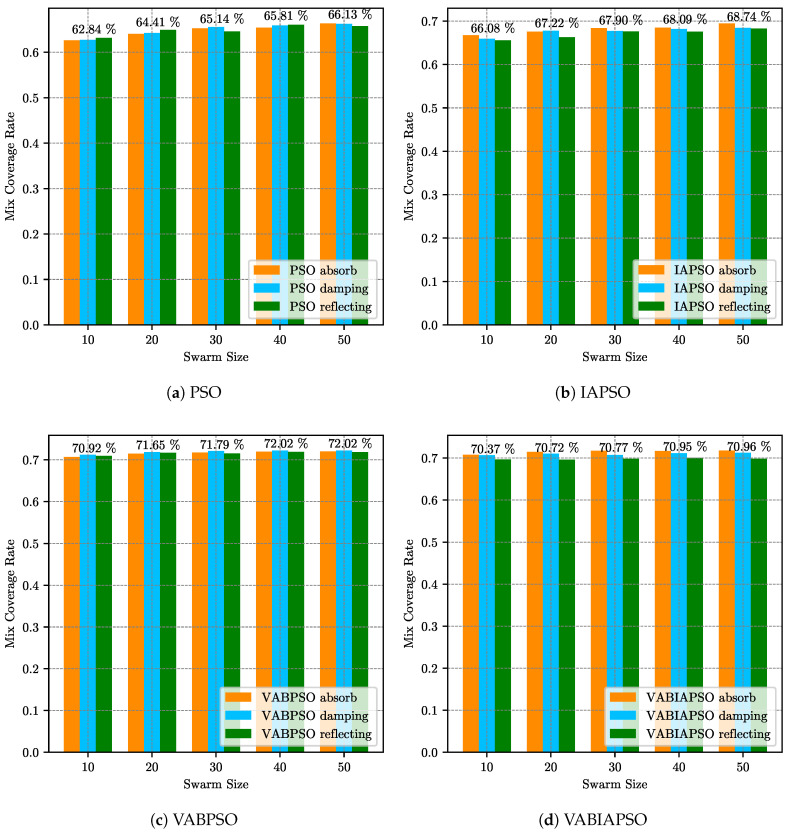
The mix coverage of the four algorithms under different swarm sizes in the real scene.

The optimization process is the same as the ideal scene. Firstly, the swarm size and boundary treatment methods of the four algorithms are determined. The experimental results are shown in Figure 10. It can be concluded that the optimal swarm size of the standard PSO algorithm is 40, and the reflecting method is the best one for dealing with boundary problems. The IAPSO algorithm needs to increase the swarm size to 50. The absorb method performs well in multiple swarm sizes. The VABPSO algorithm adopts the damping boundary processing method to reach the optimal performance when the swarm size is 20. The effect of the VABIAPSO algorithm is different from that of the ideal scene. In this case, the performance of the three boundary methods does not have much difference. The best solution can be obtained under the situation that the swarm size with the absorb method is 20.

**Figure 11:**
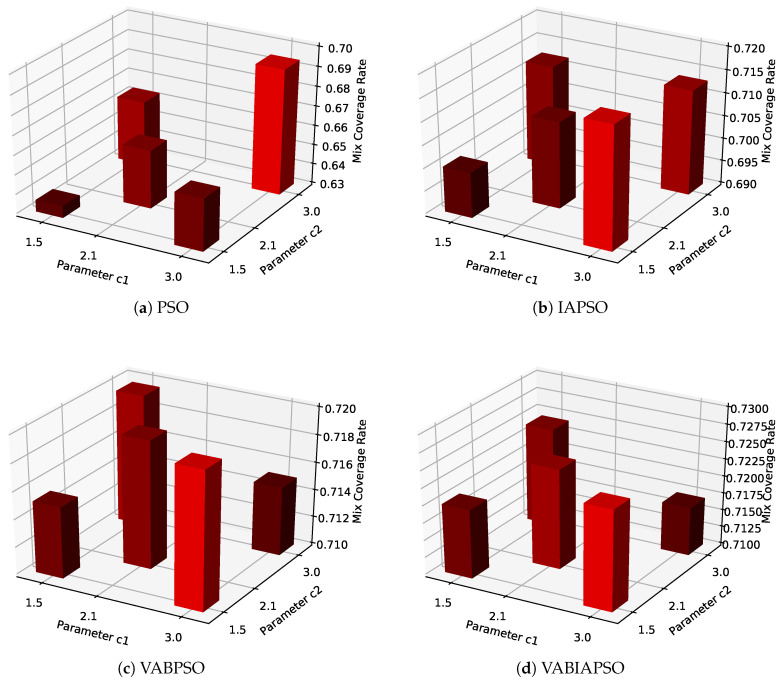
The mix coverage of the four algorithms under different values of $C_{1}$ and $C_{2}$ in the real scene.

Then, experiments with different parameters $C_{1}$ and $C_{2}$ are carried out, and the results are shown in Figure 11. The standard PSO algorithm adopts the parameter $C_{1}$ = $C_{2}$ = 3.0, which is obviously better than other combinations. However, the final mix coverage rate is still lower than the other three algorithms. The IAPSO algorithm uses $C_{1}$ = 3.0 and $C_{2}$ = 1.5 to get the best effect. In the case of three-parameter combinations, the mix coverage rates of the VABPSO and the VABIAPSO algorithms are basically the same. $C_{1}$ = 3.0, $C_{2}$ = 1.5 are the optimal parameter combinations of the two algorithms.

**Figure 12:**
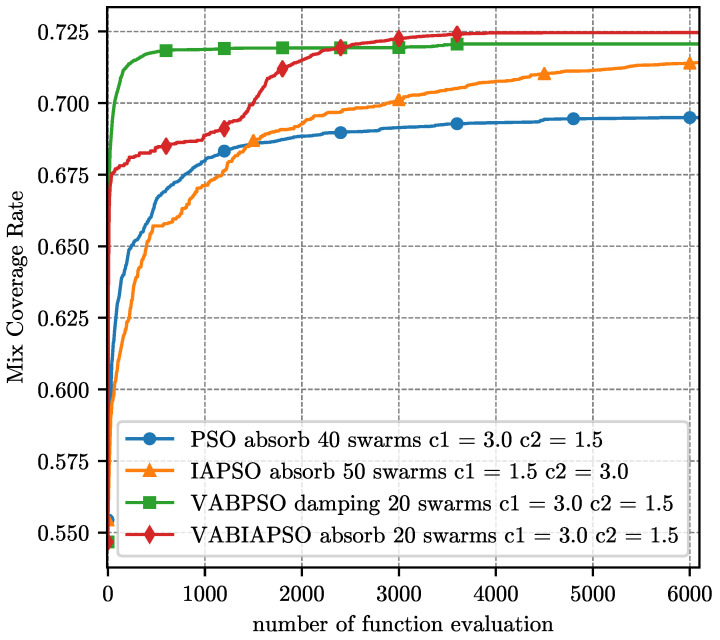
Performance comparison of four algorithms under the optimal parameters of the real scene.

The four algorithms adapt their own optimal parameters, and the comparison of the final coverage performance results in the real scene is shown in Figure 12. The difference from the ideal scene is that the mix coverage is no longer a similar result. The standard PSO algorithm has the worst effect, and the IAPSO has a significant improvement. After running 2300 operations, the VABIAPSO algorithm exceeds the VABPSO algorithm and achieved the highest mix coverage. The VABPSO algorithm has the fastest convergence speed, and the convergence is basically completed at 700 operations. The convergence speed of the IAPSO algorithm is relatively stable, and it does not converge until 6000 operations. However, the convergence sped of the VABIAPSO algorithm increases suddenly between 1300 and 1800 operations and finally converges around 4000 operations. The convergence speed of the standard PSO algorithm is very slow after 1200 operations.

**Figure 13:**
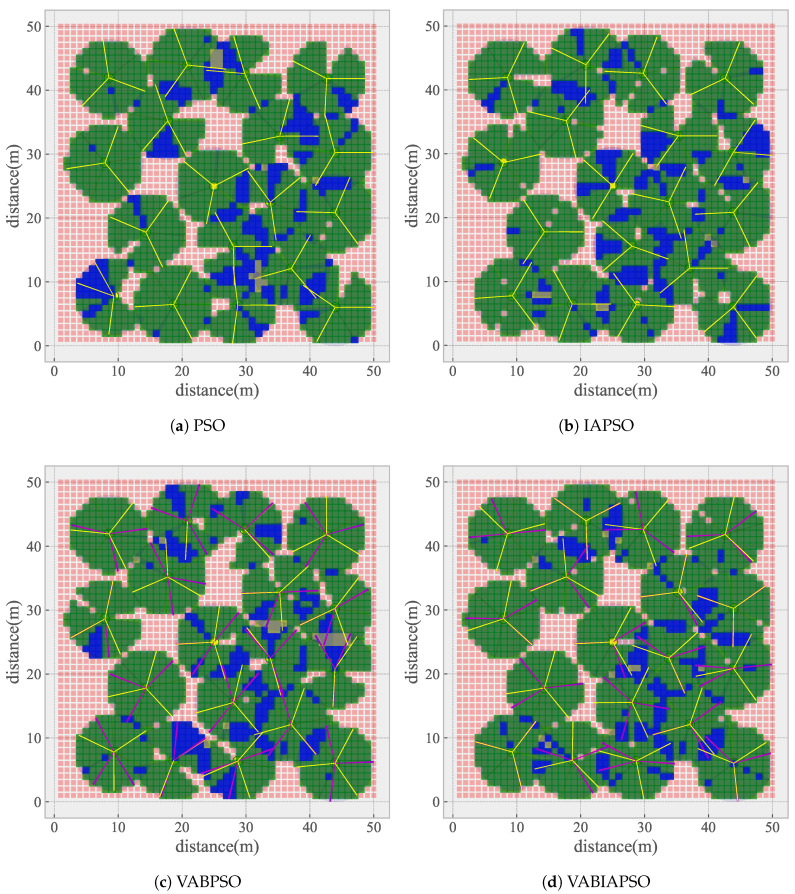
The coverage of the real scene after algorithm optimization.

The final coverage of the four algorithms in the real scene is shown in Figure 13. In Figure 13c,d, purple line segments are added to represent the boundary positions of the VABPSO and the VABIAPSO algorithms. As shown in Figure 13a, the standard PSO algorithm basically completes the Angle separation of each directed sensor on the same node. It is only the case that the two sensors are close in the lower-left corner. In addition, there is a multi-coverage in the sampling area directly above. From Figure 13b, the IAPSO algorithm solves the uncovered problem of the standard PSO algorithm in the lower-left corner, but also produces multi-coverage. The VABPSO algorithm effectively improves the search speed by adding boundary constraints that limit the search range, and the overall effect is better. Only partial multi-coverage appears on the right area of Figure 13c. The VABIAPSO algorithm combines the characteristics of two kinds of algorithms to basically achieve the best coverage effect, with less threefold coverage or more and can achieve higher coverage. The corresponding cost is that it takes a longer optimization time.

**Figure 14:**
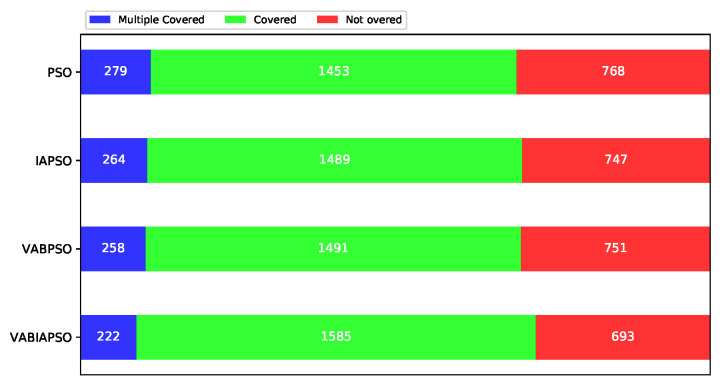
Performance comparison of four algorithms under the optimal parameters of the real scene.

The statistics of different coverage of sampling points of the four algorithms are shown in Figure 14. Blue represents the number of sampling points covered by multiple sensor nodes, green represents single coverage, and red represents not covered. Both the IAPSO and the VABPSO algorithms achieve higher coverage than the standard PSO algorithms by reducing the number of sampling points covered by multiple sensors. The VABIAPSO algorithm further reduces the proportion of multiple coverage and realizes the coverages of more sampling points at the same time.

**Figure 15:**
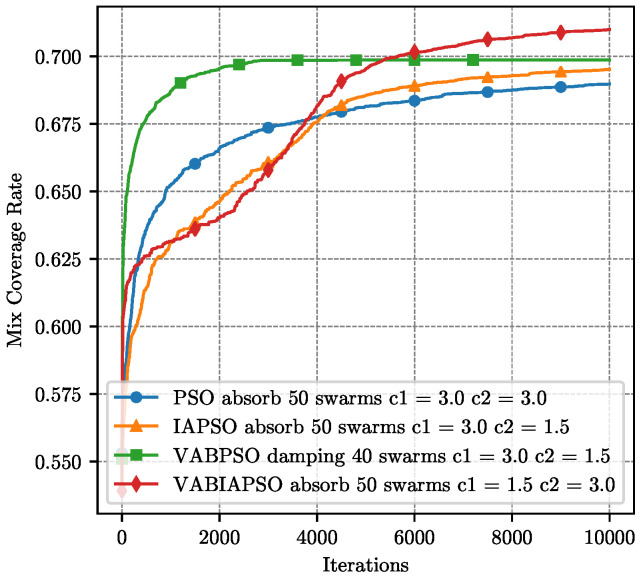
Performance comparison of four algorithms under the optimal parameters of the real scene with 4 sensors.

When the number of directional sensors on the same node is 4, we use the same optimization strategy described above. The performance comparison of four algorithms is shown in Figure 15. In the actual scenario, the optimal parameters of each algorithm are shown in the legend in Figure 15. Compared with the case of using three directional sensors, the overall coverage rate is reduced because the angle covered by each directional sensor is reduced. Considering only 4 directional sensors, the VABPSO algorithm is still the fastest algorithm with the fastest convergence rate. The IAPSO algorithm converges relatively slowly, but it has good exploration capabilities and can continuously improve the mix coverage rate. The VABIAPSO algorithm reduces part of the convergence speed and strengthens the exploration ability, reaching the maximum mix coverage rate. Experiments show that our algorithm is also applicable to a variety of sensors and has excellent performance.

**Figure 16:**
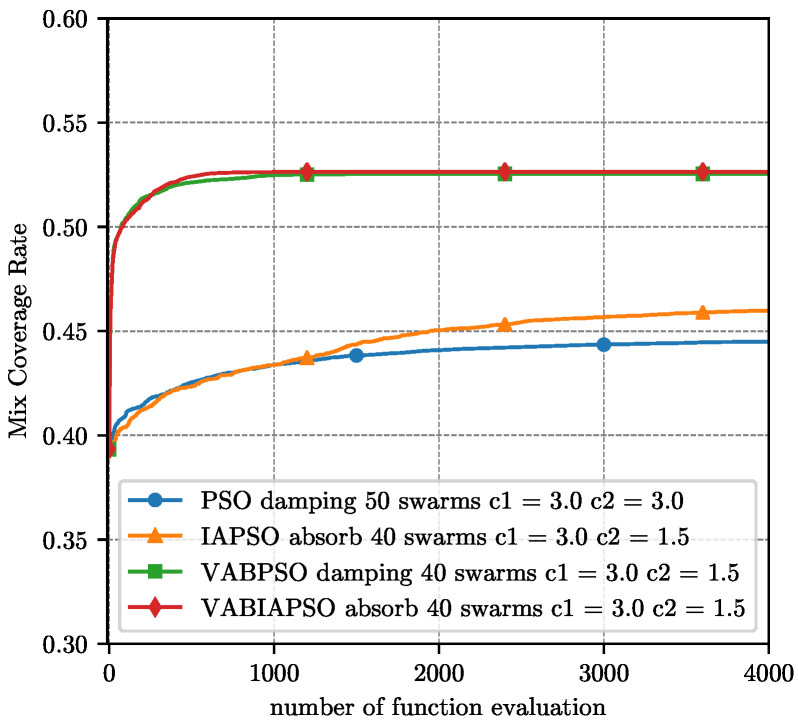
Performance comparison of four algorithms under the optimal parameters of the large-scale random scene.

Next, we apply the algorithm to a larger randomly deployed network. In this scenario, 200 nodes are randomly deployed, 3 directional sensors are installed on each node, and there are a total of 40,000 sampling points in the area. The simulation results and the optimal parameters of each algorithm are shown in Figure 16. In the simulation of large-scale networks, the effect of the algorithm using virtual angle boundary-aware is significantly better than that of the algorithm without this technology.

### 5.4. Algorithm Complexity Analysis

The algorithms using boundary constraints have advantages in coverage improvement, which is caused by the fact that these algorithms enable another kind of particle swarm to search for the optimal boundary. It improves the performance of the algorithms by occupying a part of the computing resources. Then, the algorithm complexity of the four algorithms mentioned in the paper is analyzed as follows.

The calculation of the coverage of the area is based on the coverage of each sampling point in the area, so the number of total sampling points *n* involved in the calculation should be considered. Each sampling point needs to calculate the coverage of all sensors. Sensors on a total of *m* nodes participating in the coverage. The time complexity of the algorithm is O(n×m). For the PSO algorithm, the above coverage calculation is performed for each particle in the population. For the particle swarm with the population $N_{p}$, the time complexity of the algorithm is $O(n\times m\times N_{p})$.

The IAPSO algorithm is improved based on the PSO algorithm. In addition to calculating the coverage of each point, it also calculates the multiple covers of the sampling point. The time complexity of the IAPSO algorithm is expressed as $O(\left(n+1\right)\times m\times N_{p})$. The VAB-PSO algorithm improved by the boundary constraint method is adopted to solve the problem with two-particle swarms of the same scale. One population is responsible for calculating the optimal solution, and the other is responsible for calculating the constraints of the optimal solution. Therefore, the complexity of the VAB-PSO algorithm is $O(n\times m\times N_{p}\times 2)$. Similarly, the VAB-IAPSO algorithm not only calculates the multiple covers of sampling points but also increases the particle swarm for optimal solution constraint, so the time complexity of the algorithm is $O(\left(n+1\right)\times m\times N_{p}\times 2)$. To sum up, in order of time-frequency, VAB-IAPSO > VAB-PSO > IAPSO > PSO. But in terms of the time complexity of the algorithms, they all belong to O(n).

## 6. Conclusions

The laying of wireless communication networks provides a foundation for future scenarios where things are connected. As the ends and edges of the Internet, WSNs provide massive data for the core network and reduce resource costs such as manpower and equipment. In this paper, with the isomorphism characteristic of sensors on the same node, the VAB-PSO algorithm is designed to achieve network coverage by solving the optimal azimuth and sensing distance. Firstly, the solution space of the optimization problem is bounded, then another particle swarm is used to search the boundary constraint. Finally, the joint search of BCP optimization is carried out according to the results of CSP optimization. The problems of cyclic repetition of azimuth and boundary constraints are solved, and the search of BCP is accelerated. The system simulation results show that compared with the IAPSO algorithm, the VAB-PSO algorithm achieves higher coverage of DSNs. Regarding the selection of the best location of the node and the mobility of the node, this article does not conduct research. This issue will be the focus of future research. 

## Figures and Tables

**Table 1:** Specifications of the parameters of Virtual Angle Boundary-aware Particle Swarm Optimization (VAB-PSO) algorithm.

Parameter Name	Meaning	Value
$P_{s}$	Sampling interval	1 m
*m*	Number of nodes	7, 18
α	Flare angle	[90, 120]
*R*	Sensing distance	[6, 8] m
ω	Particle weight	[0.1, 0.9]
$N_{p}$	Particle swarm number	[10, 50]
$N_{t}$	Iterations	[100, 6000]
$C_{1}$	Correction factor 1	1.5, 2.1, 3.0
$C_{2}$	Correction factor 2	1.5, 2.1, 3.0

## Data Availability

Not applicable.
